# A Network Map of FGF-1/FGFR Signaling System

**DOI:** 10.1155/2014/962962

**Published:** 2014-04-16

**Authors:** Rajesh Raju, Shyam Mohan Palapetta, Varot K. Sandhya, Apeksha Sahu, Abbas Alipoor, Lavanya Balakrishnan, Jayshree Advani, Bijesh George, K. Ramachandra Kini, N. P. Geetha, H. S. Prakash, T. S. Keshava Prasad, Yu-Jung Chang, Linyi Chen, Akhilesh Pandey, Harsha Gowda

**Affiliations:** ^1^Institute of Bioinformatics, International Tech Park, Bangalore 560066, India; ^2^Centre of Excellence in Bioinformatics, School of Life Sciences, Pondicherry University, Puducherry 605014, India; ^3^Department of Studies in Biotechnology, University of Mysore, Manasagangotri, Mysore 570006, India; ^4^Institute of Molecular Medicine, National Tsing Hua University, Hsinchu 30013, Taiwan; ^5^McKusick-Nathans Institute of Genetic Medicine, Johns Hopkins University School of Medicine, Baltimore, MD 21205, USA; ^6^Department of Biological Chemistry, Johns Hopkins University School of Medicine, Baltimore, MD 21205, USA; ^7^Department of Pathology, Johns Hopkins University School of Medicine, Baltimore, MD 21205, USA; ^8^Department of Oncology, Johns Hopkins University School of Medicine, Baltimore, MD 21205, USA

## Abstract

Fibroblast growth factor-1 (FGF-1) is a well characterized growth factor among the 22 members of the FGF superfamily in humans. It binds to all the four known FGF receptors and regulates a plethora of functions including cell growth, proliferation, migration, differentiation, and survival in different cell types. FGF-1 is involved in the regulation of diverse physiological processes such as development, angiogenesis, wound healing, adipogenesis, and neurogenesis. Deregulation of FGF-1 signaling is not only implicated in tumorigenesis but also is associated with tumor invasion and metastasis. Given the biomedical significance of FGFs and the fact that individual FGFs have different roles in diverse physiological processes, the analysis of signaling pathways induced by the binding of specific FGFs to their cognate receptors demands more focused efforts. Currently, there are no resources in the public domain that facilitate the analysis of signaling pathways induced by individual FGFs in the FGF/FGFR signaling system. Towards this, we have developed a resource of signaling reactions triggered by FGF-1/FGFR system in various cell types/tissues. The pathway data and the reaction map are made available for download in different community standard data exchange formats through NetPath and NetSlim signaling pathway resources.

## 1. Introduction

Fibroblast growth factor (FGF) superfamily consists of structurally related polypeptides most of which function through its high affinity fibroblast growth factor receptors (FGFRs). In addition to FGFRs, they also bind to heparan sulfate proteoglycans (HPSGs) and their analog, heparin. These interactions influence the stability of FGFs in the extracellular matrix and also regulate their binding and activation of FGFRs [1–9]. In humans, FGFs are encoded by 22 genes, FGF-1-14 and FGF-16-23, and are divided into 7 subfamilies. FGFs 1–10 and 16–23 are FGFR ligands, while FGFs 11–14 are intracellular FGF homologous factors which act in a receptor-independent fashion [10]. Knock-out mice of different FGFs exhibit diverse developmental and physiological disorders [11]. For instance, FGF-9 is involved in the development of lung and testes [12, 13], FGF-3 is critical for inner ear development [14], and FGF-18 is important in bone and lung development [15–17]. Moreover, knock-out of FGFs 4, 8, 9, 10, 15, 18, or 23 was found to be lethal in mice [18]. FGFs are also involved in wound healing, tissue repair [19, 20], and angiogenesis [21]. Facilitating cell proliferation, migration, and differentiation [16, 22–26], FGFs are implicated in diverse pathological conditions including cancer [27] as well as metabolic and developmental disorders [18].

Most FGFs have an N-terminal signal peptide and are thus secreted. FGFs 1, 2, 9, 16, and 20 do not have signal peptides. FGFs 9, 16, and 20 may be released through classical secretory pathway; however, FGF-1 and FGF-2 are released from damaged cells or through endoplasmic reticulum-golgi independent exocytotic pathway [10]. FGF-1 along with FGF-2 was initially isolated from bovine pituitary extracts based on their ability to induce proliferation in 3T3 fibroblasts [28, 29]. Also known as acidic FGF, FGF-1 is a 155 amino acid long non-glycosylated polypeptide. FGF-1 is not released from the cells under normal physiological conditions, but it was secreted in response to stress conditions such as heat shock, hypoxia [30, 31], serum starvation [32], and exposure to low-density lipoproteins [33]. Stress induces the release of inactive disulfide bond-linked homodimeric form of FGF-1, which is dependent on p40-Syt1, S100A13, and Cu^2+^ ions [34–37]. FGF-1 has been shown to reduce apoptosis in vascular injury [38–40]. Administration of FGF-1 has shown promise as a therapeutic strategy against human cervical spinal cord injury [41] and ischemic conditions [42–44]. Increased expression of FGF-1 was observed in ovarian [45] and prostate cancers [46]. Taken together, FGF1 is involved in different cellular functions that are mediated through its interaction with the four FGF receptors [47, 48]. A pathway resource representing these diverse functions and the underlying mechanisms that regulate these processes would be immensely useful.

Curated pathway maps are invaluable resources for scientific community. Such comprehensive pathway datasets are being increasingly used in bioinformatics efforts directed towards analysis of high-throughput datasets from various disease contexts. Repositories including Pathway Interaction Database of the National Cancer Institute (http://pid.nci.nih.gov/), Database of Cell Signaling (http://stke.sciencemag.org/cm/), KEGG Pathway Database (http://www.genome.jp/kegg/pathway.html), and INOH Pathway Database (http://inoh.org/) have cataloged basic components of FGF signaling. We have expanded the scope of this by providing a comprehensive representation of FGF1 signaling pathway and its diverse roles in regulating various cellular processes.

## 2. Methodology

Documentation of specific pathway reactions scattered in the literature into an organized, user-friendly, query-enabled platform is primary to the analysis of signaling pathways. We used NCBI PubMed database to carry out an extensive literature search to retrieve research articles where molecular events triggered by the FGF-1/FGFR signaling system were studied. Specific molecular events screened include (a) physical associations between proteins, (b) posttranslational modifications (PTMs), (c) change in subcellular localization of proteins, (d) activation or inhibition of specific proteins, and (e) regulation of gene expression. Relevant information from research articles were manually documented using the curation tool, PathBuilder. To streamline and organize data collection from literature, we followed the previously described criteria for the inclusion/exclusion of pathway specific reactions [49, 50]. The data accumulated was submitted to the NetPath signaling pathway resource developed by our group [51]. We then generated a signaling map for this pathway using PathVisio pathway visualization software. We also applied additional criteria to filter out low confidence reactions from the gathered data [52] and generated a NetSlim map. In addition to curation of molecular level information, we have also cataloged physiological effects brought about by FGF-1 in different cell types/tissues.

## 3. Results and Discussion

Canonical FGF/FGFR signaling reactions have been documented in a few public repositories and review articles. Vast amount of literature in the last few years have revealed several novel pathway intermediates of FGF/FGFR signaling system. In order to generate a comprehensive view of FGF/FGFR signaling pathway, we carried out extensive literature search on PubMed for articles pertaining to FGF-1 signaling. Of a total of 3275 articles that were screened, 237 of them had molecular reactions reported downstream of FGF-1 in various cell types/tissues. Manual curation from these research articles revealed 109 molecules involved in FGF-1 induced physical associations, modulation by PTMs, activity, and subcellular or cell surface translocation events. Of the 42 physical associations that were cataloged, 29 were “binary” and 13 were “complex” interactions inclusive of the ligand/receptor interactors. We could record a total of 87 catalysis events, 15 activation/inhibition, and 21 translocation events. The 87 catalysis events include 19 events, where the enzymes directly catalyzing the reactions were studied and reported, and 68 events for which the enzymes which post-translationally modified the proteins are not studied under FGF-1 stimulation. Apart from these molecular reactions, we have also cataloged 117 genes whose expression is reported to be either upregulated or downregulated by FGF-1 treatment. However, only a total of 25 genes were reported to be differentially regulated at mRNA level by FGF-1 stimulation in different human cell types. A list of genes reported to be regulated by FGF-1 in different mammalian systems at the mRNA and/or the protein level is provided in Table 1. After the annotation process, all the entries were reviewed and approved by internal reviewers. Internally reviewed pathways were further reviewed and approved by an external pathway authority (LC, who is an author in this paper).

### 3.1. Signaling Modules Activated by FGF-1

Signaling modules comprise a well-characterized group of molecules and their interactions downstream of activation of a receptor. We documented the following signaling modules to be activated upon stimulation with FGF-1.

#### 3.1.1. Ras/Raf/Mek/Erk Pathway

The Ras/Raf/Mek/Erk pathway has been implicated in cellular processes including cell growth, proliferation, and migration. Stimulation of different cell types with FGF-1 resulted in the formation of multiple complexes involving FRS2, GAB1, SOS1, PTPN11, SHC1, SH2B1, and GRB2 [53–60]. These complexes are critical to the subsequent activation of Ras [53, 56]. Association of Ras with Raf kinase [53] induces autophosphorylation and activation of Raf. Activation of Raf leads to phosphorylation dependent activation of Map kinases 1/2 (MAP2K1/2) and subsequently Erk2/1 (MAPK1/3) [60–62]. In the context of FGF-1 signaling, this module was reported to be involved in a number of processes including neurogenesis, adipocyte differentiation, cell proliferation, cholesterogenesis, cardioprotection, and tumor invasion and metastasis [62–67].

#### 3.1.2. Pi3k/Akt Pathway

The complexes mentioned above also lead to the activation of Pi3k/Akt pathway, another signaling module that regulates various processes including cell growth, survival, cell proliferation, and cell migration [68]. A number of studies have shown FGF-1 induced phosphorylation of Akt [63, 64, 69]. Pi3k inhibitor-based functional assays also proved the involvement of FGF-1 pathway in diverse physiological conditions including angiogenesis [70], lung development [71], maintenance of neuronal phenotype [72], neuroprotection [73], and ApoE-HDL secretion [69].

#### 3.1.3. Jnk and p38 Mapk Pathway

The c-jun N-terminal kinase** (**Jnk) pathway is implicated in the regulation of cell cycle, cell survival and apoptosis. FGF-1 stimulates the phosphorylation of p38 Mapk (MAPK14) as well as Jnk1/2 (MAPK8/9). The Jnk1/2 was also found to be crucial to neurogenesis and vascular remodeling [63, 74]. The specific functions of FGF-1 signaling mediated by p38 Mapk include growth arrest, promotion of apoptosis in response to oxidative stress, and formation of actin stress fibers [75–77].

#### 3.1.4. STAT3 and Nf-kb Pathway

FGF-1 also stimulates STATs (STAT1 and STAT3) and Nf-kB signaling modules. FGFR signaling is reported to be regulated through several downstream molecules including JAK2, SRC, SH2B1, MAPK1/3, MAPK8/9, and STAT3. This signaling axis is known to regulate various cellular processes including neurite outgrowth, cell proliferation, and increased cancer cell invasion [78–80]. In addition, FGF-1 is also reported to induce MMP9 expression in mammary adenocarcinoma cells through the Nf-kb pathway [81].

### 3.2. Physiological Effects Mediated by FGF-1

FGF-1 was found to be involved in a number of biological processes. It is associated with the development of heart [82], lens [83], lung, and liver [84–86]. Its crucial roles in neurogenesis as well as adipogenesis [65, 87, 88] have also been reported. FGF-1 induces growth arrest and differentiation in chondrocytes [89–92]. It is implicated in angiogenesis [93–95] and wound healing [95–99]. Multiple studies have also shown the role of FGF-1 in cardioprotection [99–101] and neuroprotection [22, 102]. FGF-1 also induces migration [103–105] and proliferation [106–108] in different types of cancer cells. It is also involved in the regulation of epithelial-to-mesenchymal transition [109, 110], and tumorigenesis [111] as well as invasion and metastasis [64, 112]. A list of functional effects of FGF-1 studied in different cell types/tissues is provided in Table 2.

### 3.3. Pathway Visualization, Data Formats, and Availability

**Figure 1:**
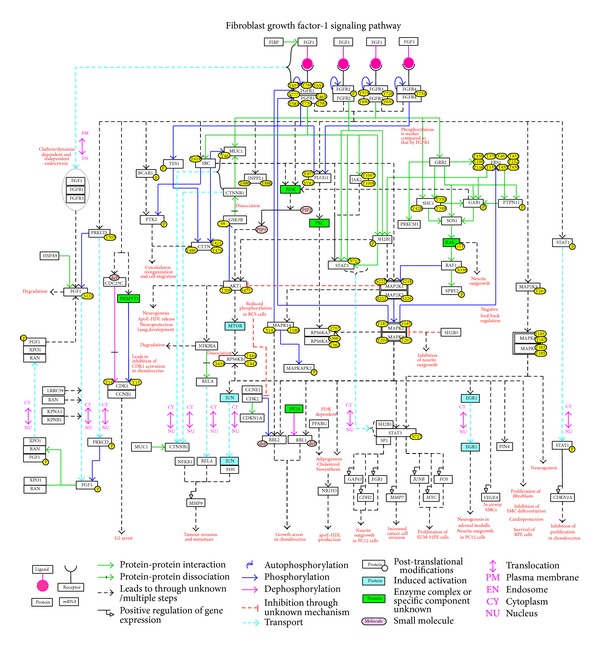
Network map of FGF-1 signaling. This map manually drawn using PathVisio [112] represents the reactions induced by FGF-1 through their receptors. Each node represents the molecules and the post-translationally modified states of proteins are also represented. Distinguished by color and continuous/dashed lines, the edges represent the specific information such as protein-protein interactions, enzyme-substrate reactions, reactions mediated through unknown/multiple steps, and protein translocations as provided in the legend. The biological processes that FGF-1 regulates through multiple signaling modules are also represented. A NetSlim [52] version of this map can be obtained from http://www.netpath.org/netslim/FGF-1_pathway.html.

User-friendly visualization of pathways is an important aspect to provide a concise view. A number of tools are available for visualization and analysis of pathway data including Cytoscape [113], ChisioBioPAX Editor (ChiBE) [114], visualization and layout services for BioPAX pathway models (VISIBIOweb) [115], and ingenuity pathway analysis. These tools use pathway and molecular interaction data in different XML-based community standard data exchange formats as input. These standard formats, which include Proteomics Standards Initiative for Molecular Interaction (PSI-MI version 2.5), Biological Pathway eXchange (BioPAX level 3), and Systems Biology Markup Language (SBML version 2.1), enable easy data exchange and interoperability with multiple software. We have provided the annotated pathway data in the standard formats mentioned above. This data can be downloaded and used from NetPath [51], an open source resource for signal transduction pathways developed by our group (http://www.netpath.org/index.html). Additionally, we have drawn a map of FGF-1/FGFR signaling using the data accumulated in NetPath. This network map represents the molecules and their reactions organized by topology and excludes the molecules identified through phosphoproteomics approaches for which topology could not be assigned (Figure 1). The map was manually drawn using freely available software, PathVisio [116]. The topology of the molecules and their reactions in the pathway was arranged based on (i) inhibitor-based assays, (ii) mutation-based assays, (iii) knock-out studies, (iv) prior knowledge of canonical modules, and/or (v) with reference to multiple review articles. Another map, which incorporated high confidence reactions in accordance with NetSlim criteria [52], is submitted to the NetSlim database. These maps can be visualized and downloaded in gpml, GenMAPP, png, and pdf formats from http://www.netpath.org/netslim/FGF-1_pathway.html. Each node in the map is linked to their molecule page in NetPath, thereby to other pathways in NetPath, and to HPRD [117] and RefSeq protein accessions. In the “map with citation” option, the edges connecting the nodes are linked to the corresponding articles in PubMed that report the FGF-1 stimulated reaction(s). Direct reactions are represented by solid edges. Indirect reactions are represented with dashed edges. The edges which represent the protein-protein interactions, enzyme-substrate reactions and translocation events are distinguished by different colors.

## 4. Conclusions

Availability of specific ligand-receptor mediated signaling data in community approved formats is crucial to the understanding of proteins and their reactions in diverse biological processes. Analysis of high-throughput data obtained from microarray- and mass spectrometry-based platforms essentially relies on enrichment of biological function or signaling pathways available in databases to obtain insights into their physiological functions. Although some resources have cataloged FGF signaling in general, this is the first attempt to provide a comprehensive view of FGF-1 signaling. This will be extended to other FGF ligands and/or specific FGFRs in the future to facilitate the analysis of differences between different FGFs and/or FGFRs. The pathway information has been made available through NetPath and NetSlim resources in multiple community standard data formats. The FGF-1 signaling pathway data will be periodically updated in NetPath. We have cataloged multiple signaling modules that are activated upon activation of FGFR and their implications in diverse physiological and pathophysiological processes. We believe that the data presented here will boost further research in this area and will help identify novel therapeutically important molecules that could be targeted in pathological conditions involving aberrant FGF-1 signaling.

## Figures and Tables

**Table 1:** List of genes that are reported to be transcriptionally and translationally regulated by FGF-1 in humans and other mammals.

	Gene symbol	Up-/down regulation	mRNA/Protein	Experiment	Organism	Tissue/cell line/type	PubMed ID	Transcriptional regulator	Regulator Gene ID	PubMed ID
1	APOE	Up	mRNA and protein	RT-PCR, Western blot	Rat	Astrocytes	18216067, 19229075, 17548887, 15627653			
2	BAMBI	Down	mRNA and protein	RT-PCR, Western blot	Human	Preadipocytes	22187378			
3	CCND1	Up	mRNA and protein	Gene chip array, Western blot	Human, rat	MG63 osteoblastic cells, Rat Wister bladder tumor cells (NBT-II)	15572039, 18189245			
4	CDK5R1	Up	mRNA and protein	Q-PCR, Western blot	Rat	PC12 cells	19249349			
5	CDKN1A	Up	mRNA and protein	RT-PCR, Western blot	Human, mouse, rat	Chondrocytes, REtsAF cells	16091747, 16153144, 11779141, 10364154	STAT1	6772	11779141, 10364154
6	CEBPA	Up	mRNA and protein	RT-PCR, Western blot	Human, mouse	Preadipocytes, 3T3-L1 cells	17068114			
7	CEBPB	Up	mRNA and protein	RT-PCR, Western blot	Human, mouse	Preadipocytes, 3T3-L1 cells	17068114			
8	COX2	Up	mRNA and protein	Northern blot, ELISA	Human, rabbit	Cardiac muscle microvessel endothelial cells	8790580, 2107185			
9	EGR1	Up	mRNA and protein	Q-PCR, Western blot	Mouse, rat	PC12 cells, Hippocampal neuronal cell line HT22, human periodontal ligament cells	19249349, 20649566, 18179472, 24396070	STAT3, SP1	6774, 6667	24396070
10	FOS	Up	mRNA and protein	RT-PCR, northern blot (mouse and rat), Immunohistochemistry, Western blot	Mouse, rat, human	3T3 cells, Adipocytes, ENU1564 cell, Astrocytes of periventricular zone of third ventricle, SUM-52PE cells	16309174, 2507555, 18041768, 11172932, 20388777			
11	JUN	Up	mRNA and protein	RT-PCR, Western blot	Rat	ENU1564 cells	18041768			
12	JUNB	Up	mRNA and protein	Gene chip array (Rat), Western blot	Rat, human	Rat Wister bladder tumor cells (NBT-II), SUM-52PE cells	18189245, 20388777			
13	MDM2	Up	mRNA and protein	RT-PCR, Western blot	Rat	REtsAF cells	16091747			
14	MMP14	Up	mRNA and protein	Northern blot, Gene chip array, Western blot	Human, rat	Prostate cancer cell line, LNCaP, Rat Wister bladder tumor cells (NBT-II)	14673954, 18189245	STAT3	6774	14673954
15	MMP9	Up	mRNA and protein	RT-PCR, Gene chip array, Western blot	Rat	ENU1564 cells, Rat Wister bladder tumor cells (NBT-II)	18041768, 18189245	RELA, JUN, FOS	5970, 3725, 2353	18041768
16	MYC	Up	mRNA and protein	Northern blot (Mouse), Western blot	Mouse, human	3T3 cells, SUM-52PE cells	16309174, 20388777			
17	NOS2	Up	mRNA and protein	RT-PCR, Western blot	Rat	Astrocytes	16524372			
18	PLAU	Up	mRNA and protein	RT-PCR, ELISA	Human	Fibroblasts	12008951			
19	PPARG	Up	mRNA and protein	RT-PCR, Western blot	Human, mouse	Preadipocytes, 3T3-L1 cells	17068114, 22187378			
20	SLC2A4	Up	mRNA and protein	RT-PCR, Western blot	Human, mouse	Preadipocytes, 3T3-L1 cells	22187378, 17068114			
21	THY1	Up	mRNA and protein	Northern blot, Western blot	Rat	PC12 cell lines	11084019			
22	TNFRSF12A	Up	mRNA and protein	RT-PCR, Immunoblot	Rat	Cardiomyocytes	19629561			
23	NGF	Up	mRNA and Protein	RT-PCR, Enzyme Immuno assay	Rat	Hippocampal astrocytes, skin fibroblasts, Primary spinal cord astrocyte	1377078, 15773903			
24	VEGFA	Up	mRNA and protein	Real time PCR, ELISA	Human	Primary human airway smooth muscle cells	22205500			
25	ACPL2	Down	mRNA	Microarray	Mouse	Osteoblast cells	18505824			
26	ARG1	Up	mRNA	Gene chip array, Q-PCR	Rat	Rat Wister bladder tumor cells (NBT-II)	18189245			
27	ATP2A2	Up	mRNA	RNA gel blot	Mouse	NIH 3T3 cells	7506544			
28	AXIN2	Down	mRNA	Microarray	Mouse	Osteoblast cells	18505824			
29	BGLAP	Up	mRNA	*in situ* hybridization	Mouse	Mouse calvaria cells (coronal sutures)	12674336			
30	CTSC	Up	mRNA	Gene chip array	Rat	Rat wister bladder tumor cells (NBT-II)	18189245			
31	DKK3	Down	mRNA	Microarray	Mouse	Osteoblast cells	18505824			
32	DLL1	Down	mRNA	Northern blot	Mouse	Neuroepithelial precursor (E10)	11466430			
33	DUSP1	Up	mRNA	Gene chip array	Rat	Rat Wister bladder tumor cells (NBT-II)	18189245			
34	DYNC2LI1	Up	mRNA	Gene chip array	Rat	Rat Wister bladder tumor cells (NBT-II)	18189245			
35	EDNRA	Up	mRNA	Northern blot	Rat	Arterial smooth muscle cells	12851419			
36	EFNB1	Up	mRNA	Gene chip array	Rat	Rat Wister bladder tumor cells (NBT-II)	18189245			
37	ELF4	Up	mRNA	Gene chip array	Rat	Rat Wister bladder tumor cells (NBT-II)	18189245			
38	FASN	Up	mRNA	RNA gel blot	Mouse	NIH 3T3 cells	7506544			
39	FGF1	Up	mRNA	RT-PCR	Rat	Pheochromocytoma cells	8576258			
40	FGF7	Up	mRNA	RT-PCR	Mouse	Embryonic lung mesenchymal cells	10446271			
41	FN1	Up	mRNA	Gene chip array	Rat	Rat Wister bladder tumor cells (NBT-II)	18189245			
42	FZD1	Down	mRNA	Microarray	Mouse	Osteoblast cells	18505824			
43	FZD2	Down	mRNA	Microarray	Mouse	Osteoblast cells	18505824			
44	FZD7	Down	mRNA	Microarray	Mouse	Osteoblast cells	18505824			
45	FZD8	Down	mRNA	Microarray	Mouse	Osteoblast cells	18505824			
46	F3	Down	mRNA	Northern blot	Human	Human umbilical vein endothelial cells	9157959			
47	GADD45A	Down	mRNA	Microarray	Mouse	Osteoblast cells	18505824			
48	HBEGF	Up	mRNA	Gene chip array	Rat	Rat Wister bladder tumor cells (NBT-II)	18189245			
49	HMGA2	Down	mRNA	Northern blot	Rat	3T3-L1 cells	10490844			
50	IBSP	Up	mRNA	*in situ* hybridization	Mouse	Mouse calvaria cells (coronal sutures)	12674336			
51	IGF1	Down	mRNA	RT-PCR	Human	Fibroblasts	12008951			
52	IGF2	Down	mRNA	RT-PCR	Human	Fibroblasts	12008951			
53	IGF1R	Down	mRNA	RT-PCR	Human	Fibroblasts	12008951			
54	IGF2R	Down	mRNA	RT-PCR	Human	Fibroblasts	12008951			
55	IGFBP4	Down	mRNA	RT-PCR	Human	Fibroblasts	12008951			
56	IL4	Up	mRNA	Q-PCR	Rat	Transected spinal cord tissue	21411654			
57	IRS1	Down	mRNA	Microarray	Mouse	Osteoblast cells	18505824			
58	LAMA3	Up	mRNA	Gene chip array	Rat	Rat Wister bladder tumor cells (NBT-II)	18189245			
59	LRRC17	Down	mRNA	Microarray	Mouse	Osteoblast cells	18505824			
60	MITF	Up	mRNA	Microarray	Mouse	Osteoblast cells	18505824			
61	MMP13	Up	mRNA	Gene chip array, Q-PCR	Rat	Rat Wister bladder tumor cells (NBT-II)	18189245			
62	MMP3	Up	mRNA	Northern blot	Rat	PC12 cell lines	11084019			
63	MSH6	Up	mRNA	RNA gel blot	Mouse	NIH 3T3 cells	8870641			
64	MSX2	Up	mRNA	*in situ* hybridization	Mouse	Mouse calvaria cells	12674336			
65	NID2	Up	mRNA	Gene chip array	Rat	Rat Wister bladder tumor cells (NBT-II)	18189245			
66	NOTCH1	Up	mRNA	Northern blot, Gene chip array, Q-PCR	Mouse, rat	Neuroepithelial precursor (E10), bladder tumor cells (NBT-II)	11466430, 18189245			
67	NR1H3	Up	mRNA	RT-PCR	Rat	Astrocytes	19229075			
68	ODC1	Up	mRNA	Northern blot	Mouse	NIH 3T3 cells	9223379			
69	PDGFA	Up	mRNA	RNA gel blot	Human	HUVE cells	1689299			
70	PFKL	Up	mRNA	RNA gel blot	Mouse	NIH 3T3 cells	7506544			
71	PLAT	Up	mRNA	RT-PCR	Human	Fibroblasts	12008951			
72	PLAUR	Up	mRNA	RT-PCR	Human	Fibroblasts	12008951			
73	PLF	Up	mRNA	Northern blot	Mouse	NIH 3T3 cells	9223379			
74	PMEPA1	Down	mRNA	Microarray	Mouse	Osteoblast cells	18505824			
75	PNRC1	Up	mRNA	Gene chip array	Rat	Rat Wister bladder tumor cells (NBT-II)	18189245			
76	POSTN	Up	mRNA	Northern blot	Rat	Pulmonary arterial smooth muscle cells	15121739			
77	PPIA	Up	mRNA	Northern blot	Rat	PC12 cell lines	11084019			
78	PRICKLE1	Down	mRNA	Microarray	Mouse	Osteoblast cells	18505824			
79	PRPH	Up	mRNA	Northern blot	Rat	PC12 cell lines	11084019			
80	PTPRE	Up	mRNA	Gene chip array	Rat	Rat Wister bladder tumor cells (NBT-II)	18189245			
81	RUNX2	Up	mRNA	*in situ* hybridization	Mouse	Mouse calvaria cells (coronal sutures)	12674336			
82	SCGB1A1	Up	mRNA	RT-PCR	Mouse	Mouse lung epithelium	12242715			
83	SDC1	Up	mRNA	Gene chip array	Rat	Rat Wister bladder tumor cells (NBT-II)	18189245			
84	SERPINB1	Down	mRNA	Microarray	Mouse	Osteoblast cells	18505824			
85	SERPINB2	Up	mRNA	RT-PCR	Human	Fibroblasts	12008951			
86	SERPINE1	Up	mRNA	RT-PCR	Human	Fibroblasts	12008951			
87	SFRP1	Down	mRNA	Microarray	Mouse	Osteoblast cells	18505824			
88	SFTPC	Up	mRNA	RT-PCR	Mouse	Mouse lung epithelium, Embryonic stem cell (mESC) line E14-Tg2a	12242715, 20497026			
89	SOCS1	Up	mRNA	Northern blot	Rat	Mouse lens epithelium	14985304			
90	SOCS3	Up	mRNA	Northern blot	Rat	Mouse lens epithelium	14985304			
91	SOX2	Up	mRNA	Microarray	Mouse	Osteoblast cells	18505824			
92	SPP1	Up	mRNA	Quantitative northern blot	Rat	Pulmonary arterial smooth muscle cells	15121739			
93	SPRY1	Up	mRNA	RNA gel blot	Mouse	MC3T3-E1 osteoblasts	16604287			
94	SPRY2	Up	mRNA	RNA gel blot	Mouse	MC3T3-E1 osteoblasts	16604287			
95	SPRY4	Up	mRNA	RNA gel blot	Mouse	MC3T3-E1 osteoblasts	16604287			
96	S1PR3	Up	mRNA	Northern blot	Human	Human umbilical vein endothelial cells	9315732			
97	TCF3	Down	mRNA	Microarray	Mouse	Osteoblast cells	18505824			
98	TCF4	Down	mRNA	RT-PCR	Human	Preadipocytes	22187378			
99	TGFA	Up	mRNA	Northern blot	Mouse	Cultured keratinocytes	7535082			
100	TGFB2	Down	mRNA	Microarray	Mouse	Osteoblast cells	18505824			
101	TGFBR3	Down	mRNA	Microarray	Mouse	Osteoblast cells	18505824			
102	THBS1	Down	mRNA	Microarray	Mouse	Osteoblast cells	18505824			
103	THBS1	Up	mRNA	Northern blot	Mouse	NIH 3T3 cells	9223379			
104	TIMP1	Up	mRNA	Gene chip array	Rat	Rat Wister bladder tumor cells (NBT-II)	18189245			
105	TIMP3	Down	mRNA	Microarray	Mouse	Osteoblast cells	18505824			
106	VIM	Up	mRNA	Gene chip array	Rat	Rat Wister bladder tumor cells (NBT-II)	18189245			
107	ADIPOQ	Up	Protein	Radioimmunoassay	Human	Preadipocytes	17068114			
108	CCNE1	Up	Protein	Western blot	Human	MG63 osteoblastic cells	15572039			
109	CTNNB1	Down	Protein	Western blot	Human	Simpson Golabi Behmel syndrome (SGBS), Preadipocytes	22187378			
110	HMOX1	Up	Protein	Western blot	Human	Spinal cord astrocytes	16524372			
111	MMP7	Up	Protein	ELISA	Human	LNCaP cells	11922392	STAT3	6774	11922392
112	PKMYT1	Up	Protein	Immunoblot	Rat	Chondrosarcoma cells	21051949			
113	PLIN1	Up	Protein	Western blot	Human, mouse	Preadipocytes, 3T3-L1 cells	17068114			
114	PTGIS	Down	Protein	ELISA	Human	Endothelial cells	2107185			
115	PTGS2	Down	Protein	ELISA	Human	Endothelial cells	2107185			
116	RELA	Up	Protein	Western blot	Rat	ENU1564 cells	18041768			
117	RHOA	Up	Protein	Immunoblot	Rat	Cardiomyocytes	19629561			
118	SOX9	Up	Protein	Western blot	Mouse	Costal chondrocytes	10655493			
119	WEE1	Up	Protein	Immunoblot	Rat	Chondrosarcoma cells	21051949			
120	CDH2	Up	Protein	Western blot	Rat	PC12 cells	24396070	STAT3, SP1	6774, 6667	24396070
121	GAP43	Up	Protein	Western blot	Rat	PC12 cells	24396070	STAT3	6774	24396070

**Table 2:** Functions of FGF-1 identified in diverse cell/tissue types of human and other mammalian origins.

Function	PubMed ID	Cell type/tissue	Organism
Adipogenesis	22187378, 17068114	Preadipocytes	Human
Apoptosis	20657013	Hepatoma cells, HEK293 cells	Human
	15773903	Motor neuron	Rat
	9681989	Peroxynitrite-induced apoptosis in PC12 cells	Rat
Cell cycle arrest	16153144	cells	Human
Cell migration	9108375	Skin fibroblasts	Human
	11019781	Fibroblasts	Mouse
Cell proliferation	9182757	Embryo fibroblasts	Rat
	2441696	Arterial smooth muscle cells	Human
	14966081	AT2 alveolar cells	Human
	15094393	Human long-bone growth plate chondrocytes	Human
	1699952	Umbilical vein endothelial ceils	Human
	15767480	Y79 cells	Human
	2303528	Epidermal keratinocytes (BALB-MK1)	Mouse
	2303528	Keratinocytes (BALB/MK-1)	Mouse
	2383402	Leydig cells (TM3)	Mouse
	1379845	Megakaryocyte progenitor cells	Mouse
	1379845	Megakaryocytes	Mouse
	14985304	Murine lens epithelial cell lines CRLE2, 1AMLE6, TN4-1 and NKR11	Mouse
	15574884	NIH-3T3 cells	Mouse
	3272188	Adrenal chromaffin cells	Rat
	2566605	Astroblasts	Rat
	1377078	Hippocampal astrocytes	Rat
	2153969	Rat bladder carcinoma cell line (NBT-II)	Rat
	8622701	PC12 cells	Rat
	8732667	Prostate cancer cells	Rat
	1638984	Retinal cells	Rat
	1377078	Skin fibroblasts	Rat
	12907464	Aortic smooth muscle cells	Human, rat
	1638984	Retinal cells	Rats
	22108586	Periodontal fibroblasts	Rat
	3272188	Adrenal chromaffin cells	Rat
	22108586	Periodontal ligament fibroblasts	Rat
	20388777	SUM-52PE cells	Human
Cell rounding, growth inhibition	11779141	ATDC5 cells, chondroprogenitor cell lines	Mouse
Cholesterol biosynthesis	19713443	Mouse fibroblasts and rat astrocytes	Mouse, rat
	19229075	Astrocytes	Rat
	18216067	Astrocytes	Rat
	17548887	Astrocytes	Rat
Differentiation	20497026	Embryonic stem cell (mESC) line E14-Tg2a	Mouse
Epithelial-mesenchymal transition	2153969	NBT-II cells (Rat bladder carcinoma cell line)	Rat
	7593195	NBT-II	Rat
	2153969	NBT-II	Rat
Fiber cell differentiation	7539358	Lens epithelial cells	Mouse
G0/G1 arrest	21051949	Chondrosarcoma cells	Rat
G2 arrest	21051949	Chondrosarcoma cells	Rat
G2/M transition	20044603	Breast cancer cells	Human
Growth arrest	14593093	Rat chondrosarcoma (RCS) cells	Rat
Inhibition of apoptosis	16524372	Astrocytes	Rat
	17473910, 16091747	PC12 and RetsAF cells	Rat
Inhibition of cell growth	17363592	TAKA-1 cells	Hamster
Inhibition of neurogenesis	11466430	NEP cells	Mouse
Inhibition of proliferation	10364154	Chondrosarcoma cells (RCS)	Rat
Membrane ruffling	7534069	Human ductal breast epithelial tumor cell line (T47D)	Human
Neurite outgrowth	20175207	TREX 293 cells	Human
	3272188	Adrenal chromaffin cells	Rat
	8764646	PC12 cells	Rat
	19249349	PC12 cells	Rat
	3316527, 8576258	PC12 cells	Rat
	12127979, 9182757, 2157719	PC12 cells	Rat
Neuronal differentiation	16716298	Primary astrocyte from human fetal brain	Human
	7514169, 8622701, 2157719	PC12 cells	Rat
Osteoblast proliferation	18041768	ENU1564 cells	Rat
Osteoblast differentiation	18505824	Osteoblasts	Mouse
Osteogenic differentiation	12674336	Sutural mesenchyme in mouse calvaria	Mouse
Protection from apoptosis	19765618, 8576258	PC12 cells	Rat
Repression of myogenic differentiation	1379245	Skeletal muscle myoblasts (MM14)	Mouse
Retinal cell proliferation	15978261	Retinal cells	Mouse
Skeletal muscle development	8601591	Skeletal muscle myoblasts (MM14)	Mouse
Synaptic plasticity	20649566	Hippocampal neuronal cell line HT22	Mouse
Tumorigenesis	20889570	JMSU1 urothelial carcinoma cell lines	Human
	9038374	NBD-II	Rat
Vascular remodeling	15121739	Pulmonary arterial smooth muscle cells (PASMCs)	Rat
	22205500	ASM (Airway Smooth Muscle cells)	Human
Regeneration	3353388	Retinal ganglion cells	Rat
Astrocyte activation	15773903	Primary spinal cord astrocyte	Rat
Neurogenesis	20429889	Embryonic stem cells	Mouse
Wound healing	9036931		Mouse
Cord Formation	16631103		Rat
Decrease in food intake	7692459		Rat
Facilitation of memory	7692459		Rat
Increase in sleep duration	8985960		Rabbit
Maintenance of the integrity of the organ of corti, initiation of protective recovery and repair processes following damaging auditory stimuli	7568115		Rat
Arteriole dilation	8853345		Rat
Feeding suppressor function	11172932		Rat
Hair-cell innervation during the terminal development of the sensory epithelium	12792312		Rat
Lens regeneration	3792708		Bovine
Lung morphogenesis and differentiation	12242715		Rat
Metastasis	1707175		Rat
Muscle regeneration	1384586		Mouse
Myocardial remodeling	19629561		Rat
Neuroprotection	12095987		Rat
Prevention of premature angiogenesis and inflammatory responses	17643421		Mouse
Protection against hypoxic-ischemic injury	16635575		Rat
Spinal cord injury repair	21411654		Rat
Cardioprotection	15337227, 12176126		Mouse

## Abbreviations

S100A13: S100 calcium binding protein A13
FRS2: Fibroblast growth factor receptor substrate 2
GAB1: GRB2-associated binding protein 1
SOS1: Son of sevenless homolog 1
PTPN11: Protein tyrosine phosphatase, non-receptor type 11
SHC1: Src homology 2 domain containing transforming protein 1
GRB2: Growth factor receptor-bound protein 2
Mapk: Mitogen activated protein kinase
Pi3k: Phosphatidylinositide 3-kinase
Akt: v-akt murine thymoma viral oncogene homolog
HDL: High density lipoprotein
Jnk: Jun N-terminal kinase
STAT3: Signal transducer and activator of transcription 3.
